# Ileal digestibility of intrinsically labeled hen's egg and meat protein determined with the dual stable isotope tracer method in Indian adults

**DOI:** 10.1093/ajcn/nqy178

**Published:** 2018-10-01

**Authors:** Sindhu Kashyap, Nirupama Shivakumar, Aneesia Varkey, Rajendran Duraisamy, Tinku Thomas, Thomas Preston, Sarita Devi, Anura V Kurpad

**Affiliations:** 1 Division of Nutrition, St. John's Medical College, St. John's National Academy of Health Sciences, Bangalore, India; 2 Departments of Physiology, St. John's Medical College, St. John's National Academy of Health Sciences, Bangalore, India; 3 Departments of Biostatistics, St. John's Medical College, St. John's National Academy of Health Sciences, Bangalore, India; 4 Indian Council of Agricultural Research-National Institute of Animal Nutrition and Physiology, Adugodi, Bangalore, India; 5 Scottish Universities Environmental Research Centre, East Kilbride, Scotland, United Kingdom

**Keywords:** intrinsic labeling, egg protein, meat protein, dual stable isotope tracer method, kynurenine-tryptophan ratio, environmental enteric dysfunction, Digestible Indispensable Amino Acid Score (DIAAS)

## Abstract

**Background:**

Protein quality assessment through the Digestible Indispensable Amino Acid Score requires accurate measurements of true ileal protein and amino acid digestibility, for which a dual isotope technique was recently developed. However, the ileal digestibility of indispensable amino acids (IAA) in humans from high-quality proteins is not well known.

**Objective:**

The aim of this study was to intrinsically label hen's egg and meat protein by the use of uniformly ^2^H-labeled amino acids, and to measure their true ileal indispensable amino acid (IAA) digestibility via the dual isotope method in humans.

**Design:**

^2^H-labeled lyophilized boiled egg white protein, whole boiled egg, and cooked meat were obtained from layer hens (BV-300) administered a uniformly ^2^H-labeled amino acid mix orally for 35 d with their daily feed. The ileal IAA digestibility of these proteins was determined with reference to digestibility of previously characterized [U-^13^C]spirulina in a dual tracer method in healthy Indian subjects whose intestinal health was measured by the plasma kynurenine-to-tryptophan (KT) ratio.

**Results:**

All subjects had normal KT ratios. The mean ± SD true ileal IAA digestibility of ^2^H-labeled egg white protein, whole boiled egg, and cooked meat was 86.3% ± 4.6%, 89.4% ± 4.5%, and 92.0% ± 2.8%, respectively. Leucine digestibility correlated with the KT ratio (*r* = −0.772; *P* = 0.009).

**Conclusions:**

Uniformly ^2^H-labeled hen's egg and meat protein can be used to measure ileal IAA digestibility by the dual isotope tracer approach in humans. The mean IAA digestibility values for these high-quality proteins in the healthy Indians studied were similar to values obtained in earlier human and animal experiments. Leucine digestibility in these meal matrices correlated with the KT ratio, but this aspect needs further evaluation. This trial was registered at the Clinical Trials Registry of India (http://ctri.nic.in) as CTRI/2018/03/012265.

## INTRODUCTION

Recent recommendations have emphasized the importance of dietary protein quality and the need for its assessment by use of the Digestible Indispensable Amino Acid Score, necessitating true ileal digestibility measurements on individual indispensable amino acids (IAAs) with respect to a nutritional reference, such as milk or egg white protein (1). Whereas direct ileal balance measurements have been used to define ileal protein and amino acid digestibility in animals and humans (ileostomates and nasoileal intubation methods), isotopic methods such as the indicator amino acid oxidation method have been used to measure the metabolic availability of specific IAAs, one IAA at a time (2, 3). Recently, a dual isotope method for measuring the human ileal digestibility of different IAAs in intrinsically labeled protein was proposed (4), in which the measurement of absorbed amino acids from a ^2^H intrinsically labeled test protein could be compared against that for a ^13^C intrinsically labeled standard protein with known digestibility. It is particularly suited for measurements in vulnerable age groups and the study of varying physiological states. This method has been used to determine the digestibility of ^13^C intrinsically labeled spirulina protein with respect to that of crystalline amino acids, as well as ^2^H intrinsically labeled legume proteins in apparently healthy adults (5).

It is not known if protein digestibility varies with the presence of different environmental stressors. Although the daily IAA requirement of healthy humans has been shown to be similar globally (6–11), there has been some doubt about the adequacy of the protein requirement related to its digestibility and composition in environments where environmental enteric dysfunction (EED) with impaired small intestinal function may be widely prevalent (12). Impaired protein digestibility might be significant in India, where otherwise healthy Indian adults and children have been shown to have greater intestinal permeability, in terms of reduced mannitol absorption and higher lactulose recoveries respectively, when compared with relatively healthy subjects with the dual sugar absorption test (13, 14). However, protein digestion has seen little attention in the context of gut dysfunction.

As a start, the digestibility of high-quality proteins, such as egg, meat, or milk protein, needs to be assessed in healthy groups living in developing countries where EED might be prevalent. Egg protein is a high-quality protein source owing to its amino acid composition (6) and its high digestibility as measured by orofecal nitrogen balance (15). The ileal digestibility of leucine in boiled egg protein has been shown to be high in human ileostomates (16, 17). This study aimed to use the dual isotope method to determine the true ileal IAA digestibility of hen's egg and meat protein in healthy urban Indian men and women from middle-class environments by intrinsically labeling the hen's egg and meat protein with a low dose of uniformly ^2^H-labeled amino acids. It also aimed to compare their gut health, mainly the intestinal inflammatory status, by use of the plasma kynurenine-to-tryptophan (KT) ratio (18), with IAA digestibility.

## METHODS

### Intrinsic labeling of hen's egg and meat

We housed 2 layer hens (BV-300, 29 wk old, weight: 1.5 kg) in the Central Animal House, St. John's Medical College, in individual grated stainless-steel cages with a sloping base that allowed for easy egg collection. A lighting schedule of 16.5 h of light/d and a temperature of 28°C ± 2°C was maintained. In order to allow for acclimatization to the place and feed, the hens were provided with their regular diet before starting the actual experimental protocol. The hens were fed the same feed in continuation of their previous diet, to meet their daily energy and protein requirement, which contained 2.5 MJ metabolizable energy/kg dry matter, 16.5% protein, and 4.9% crude fiber. The amount of feed required was assessed during this period (110–130 g/d) and was accordingly administered to avoid nutritional stress and impaired egg production. Water was provided ad libitum for the entire study duration.

An Institutional Animal Ethical Committee approval was obtained for the experiment and chicken-handling procedures. First, a pilot isotope dosing study was conducted to evaluate the dose required to achieve a target enrichment of 1000 parts per million excess (ppme) in the egg white protein, assuming ∼20% transfer efficiency (19) and with the knowledge that the tracer source was chosen on economic grounds and contained an amino acid profile that differed from the amino acid profile of the feed. The pilot dose used was set at 6 mg daily of [U-^2^H]-labeled crystalline amino acid mix (DLM-6819-1, Cambridge Isotope Laboratories; >97% purity, amino acid composition provided in **Table 1**). One hen received this dose dissolved in water orally with a sterile syringe, daily for 17 d. The egg white protein mean ^2^H IAA enrichment achieved with this isotope dose was 425 ppme. Therefore, to achieve the target enrichment of 1000 ppme, both hens subsequently received 12 mg of the same mix for 35 d. Any spillage of the isotope solution during administration was recorded. Eggs were collected every day and stored at 4°C for further processing.

**TABLE 1:** Composition of [U-^2^H]-labeled crystalline amino acid mix

Amino acids	Concentration, %
l-Lysine:2HCl	12
l-Leucine	9
l-Glutamic acid	9
l-Aspartic acid	8
l-Alanine	6
l-Arginine:HCl	6
l-Glutamine	5
Glycine	5
l-Asparagine:H_2_O	5
l-Proline	5
l-Phenylalanine	4
l-Valine	4
l-Serine	4
l-Threonine	4
l-Tyrosine	3
l-Isoleucine	3
l-Tryptophan	3
l-Cysteine	3
l-Histidine:HCl:H_2_O	1
l-Methionine	1

The eggs were processed in 2 ways, to obtain either a boiled lyophilized egg white protein or a whole boiled egg. The analysis of the IAA enrichment in the egg white protein was conducted in batches of eggs collected at 3-d intervals. For the lyophilized egg white protein, eggs were boiled for 20 min in boiling water, following which the yolk and the egg white were separated under sterile conditions, minced, lyophilized, ground into a homogeneous powder with a pestle and mortar, and stored at 4°C. The lyophilized egg white protein obtained was then pooled for use in the human protocol. The same process of boiling was used for whole egg that was shelled and immediately stored at −80°C. To obtain labeled skeletal muscle, the hens were killed a day after the last isotope dosing. The meat (breast, wing, thigh, and drumstick) was pooled for each hen separately and pressure cooked for 40 min, portioned into sterile plastic containers and stored immediately at −80°C. The analysis of ^2^H isotopic enrichment of individual amino acids in the lyophilized egg white, whole egg, and meat samples was performed by liquid chromatography-tandem mass spectrometry (LC-MS/MS; 6495 Triple iFunnel Quadrupole, Agilent) following hydrolysis, cation exchange purification, and derivatization as ethoxycarbonyl ethyl esters, as described elsewhere (5).

### Human digestibility study protocol, the dual stable isotope tracer approach

A culturally acceptable test meal (ghee rice and curry with egg or meat) was standardized and prepared in batches for the egg white protein, whole boiled egg, and cooked meat protein digestibility experiments. The meal provided one-third of the daily energy and protein requirement of healthy adults matched for age and sex (the nutrient composition of the test meals is provided in **Table 2**). The labeled protein comprised two-thirds of the total protein in the meal, with the rest coming from other protein sources (rice) and with a trace amount (∼ 2%) contributed by [U-^13^C]spirulina. The whole boiled egg and cooked meat were thawed overnight in a refrigerator at 4°C. When preparing the meal, the egg white protein was mixed into the curry portion, whereas the whole boiled egg and cooked meat were added to each meal portion separately and mixed. The meals were warmed for 10 s in a microwave oven before administering it to the subjects. [U-^13^C]Spirulina (12 mg/kg; Cambridge Isotope Laboratories; 97% purity) was added to the meal as a standard with predetermined true ileal IAA digestibility, expressed with respect to crystalline amino acids (5).

**TABLE 2:** Nutrient composition of the standardized test meal for each test protein group^1^

	EWP	WBE	CM
Energy, kcal/meal	731.30 ± 72.0	717.94 ± 49.9	714.19 ± 125.8
Protein, g/meal	22.50 ± 1.8	22.08 ± 0.8	22.15 ± 3.8
Fat, g/meal	29.86 ± 1.7	30.46 ± 1.2	29.96 ± 3.5
Carbohydrate, g/meal	91.5 ± 12.1	88.14 ± 9.2	88.1 ± 19.4
P/E ratio	12.33 ± 0.3	12.33 ± 0.6	12.42 ± 0.2

^1^Values are means ± SDs, *n* = 6/group. Different subjects were used for each experiment. CM, cooked meat; EWP, egg white protein; P/E, Protein/Energy; WBE, whole boiled egg

Healthy nonsmoking subjects (*n* = 6 for each test protein group) of both sexes (equally represented), with BMI (in kg/m^2^) between 18.5 and 25, age between 18 and 45 y, and no food allergies, were included in the study. Subjects with a history of serious illness within 3 mo of the study, taking antibiotics within 4 wk prior to the study, or taking iron supplements, and those who had consumed alcohol in the 24 h prior to the study day, were excluded. All subjects gave informed written consent, and the Institutional Ethical Review Board approved the study. Subject screening and enrollment details are given in **Supplemental Figure 1**.

On the day of the experiment, subjects reported at 0630 to the metabolic unit after an overnight fast of 12 h. The experiment started at 0700 and continued for the next 8 h. The subjects were restricted to minimal physical activity during the experiment. A plateau feeding protocol was adopted. Briefly, the test meal was cooked and portioned into 11 parts, each part constituting one mini meal. The priming meal (3 mini meals), along with [^13^C]bicarbonate (3 mg/kg, Cambridge Isotope Laboratories; >99% purity), was administered first, followed by a single mini meal every hour for the next 7 h. One of the mini meal portions was retained for isotopic analysis. Breath samples were collected in a 10-mL glass evacuated tube (Becton Dickinson), whereby the subjects were instructed to blow into the tube with a single breath. The breath sample was obtained at baseline, followed by hourly samples for the total experimental duration. The samples were stored at room temperature until analysis.

Blood was collected after securing an indwelling venous catheter at the beginning of the experiment. A basal blood sample was collected followed by half-hourly samples from hour 5 onwards, until the end of the experiment. The time points for blood collection were chosen based on previous experiments to determine the appropriate sampling time points (5), as these demonstrated a plateau for the isotopic enrichment of plasma amino acids. Whole blood was transferred into EDTA-coated anticoagulant evacuated tubes (Becton Dickinson) and centrifuged at 4°C to separate the plasma, which was divided into aliquots and stored at −80°C until analysis.

Plasma samples were processed to remove proteins and purify amino acids by cation exchange, and derivatized before IAA analysis at St. John's Research Institute, by LC-MS/MS, to measure ^13^C and ^2^H isotopic enrichments of the IAA, as explained elsewhere (5). Whole meal samples underwent gas phase acid hydrolysis prior to amino acid analysis (5).

The true ileal digestibility percentage for each IAA was calculated from the equation:

(1) \begin{eqnarray*}
\frac{{\left[ {{\rm{Plasma}}{{\rm{ }}^2}{\rm{H}}\,{\rm{IAA}}\left( {{\rm{ppme}}} \right)/{\rm{Meal}}{\,^2}{\rm{H}}\,{\rm{IAA}}\left( {{\rm{ppme}}} \right)} \right]}}{{\left[ {{\rm{Plasma}}{\,^{13}}{\rm{C}}\,{\rm {IAA}}\left( {{\rm{ppme}}} \right)/{\rm{Meal}}{\,^{13}}{\rm{C}}\,{\rm{IAA}}\left( {{\rm{ppme}}} \right)} \right]}}*100*{\rm{Di}}{{\rm{g}}_{{\rm{Std}}}}/100
\end{eqnarray*}

where Dig_Std_ is the percentage true ileal digestibility of IAA of spirulina protein with reference to crystalline AA. The mean value of the IAA digestibility of spirulina, which was the standard protein, was 85.2%, and the Dig_Std_ used for each amino acid was 84.1%, 95.3%, 82.5%, 77.5%, 86.0%, 84.2%, and 87.1% for methionine, phenylalanine, threonine, lysine, leucine, isoleucine, and valine, respectively (5).

Intestinal health was estimated from the plasma KT ratio. A dansyl [5-(dimethylamino)-1-napthalene sulfonamide] derivatization technique (20) was used for the measurement of plasma tryptophan and kynurenine in the fasted state. Derivatives were analyzed by reverse-phase LC-MS/MS at St. John's Research Institute. In order to define intestinal dysfunction, the diagnostic cutoff value of kynurenine and the KT ratio was derived from the distribution of values in a healthy US adult population (21). A plasma kynurenine concentration of >4.96 μmol/L (based on the mean + 2 SD from the reported distribution of 2.40 ± 1.28 μmol/L) and KT ratio of >0.047 (calculated as the ratio of the mean + 2 SD values of kynurenine and tryptophan from the same population) was considered to be indicative of the presence of intestinal dysfunction.

Data are reported as means ± SDs. To test for differences in IAA digestibility between the protein sources, digestibility values for each IAA across the 3 test proteins were first checked for normality by use of the Shapiro-Wilk test; IAA digestibility values that were not normally distributed (threonine and valine) were log transformed for further analysis. A multivariate ANOVA was performed to determine differences in IAA digestibility between the 3 test protein groups. Adjusted Bonferroni post hoc tests were performed for pairwise comparison for groups. Pearson's correlation was performed to evaluate if potentially altered intestinal function as measured by the KT ratio and plasma concentration of kynurenine could alter IAA digestibility across all subjects in a pooled sample (since the subjects were different for each protein evaluation, and all proteins could be considered in the same class of high-quality protein). Differences and correlations were considered statistically significant if *P* < 0.05. All calculations were performed with STATA, version 15.1.

## RESULTS

### Hen's egg yield and enrichments

A total of 85 labeled eggs were collected during the experiment; eggs laid in the first 3 d were not used for the human study. A total of 156 g of dry protein was obtained from the lyophilized egg whites (39 eggs), from both the pilot and the actual experiment. The IAA enrichment of egg white tracer of the initial parts of the pilot study with the 6 mg tracer dose and the 12-mg IAA tracer dose (which confirmed the achievement of the target enrichment) is shown in **Supplemental Figure 2**. The mean ^2^H IAA enrichment for the pooled egg white protein was 1093 ppme. The total meat yield was 545 g from both hens, with mean ^2^H IAA enrichment of 828 ppme.

### True ileal protein digestibility of hen's egg and meat

The subject details on age, anthropometry, hemoglobin, plasma tryptophan, kynurenine, and KT ratios are provided in **Table 3**. None of the subjects were undernourished or overweight as defined by their BMI, and all were in good health, with no acute or chronic history of impaired gastrointestinal symptoms in the months prior to the study. Their plasma tryptophan, kynurenine, and KT ratio were in the normal range based on the values derived from the healthy US population.

**TABLE 3:** Demographic and anthropometric characteristics of study participants in each test protein group^1^

Variables	EWP	WBE	CM
Age, y	21.67 ± 4.5	23.40 ± 2.6	22.00 ± 3.4
Weight, kg	55.99 ± 3.3	55.22 ± 2.8	56.10 ± 9.1
Height, m	1.65 ± 0.1	1.60 ± 0.1	1.62 ± 0.1
BMI, kg/m^2^	20.66 ± 0.8	21.67 ± 2.4	21.21 ± 1.3
Hemoglobin, g/dL	14.53 ± 1.3	13.34 ± 1.4	13.80 ± 3.1
Plasma tryptophan,^2^ μmol/L	108.33 ± 14.4	96.50 ± 15.5	108.38 ± 24.5
Plasma kynurenine,^2^ μmol/L	2.28 ± 0.5	1.95 ± 0.6	1.93 ± 0.8
KT ratio	0.022 ± 0.006	0.020 ± 0.003	0.018 ± 0.006

^1^Values are means ± SDs, *n* = 6/group. Different subjects were used for each experiment. CM, cooked meat; EWP, egg white protein; KT, kynurenine-to-tryptophan; WBE, whole boiled egg.

^2^Fasting.

**FIGURE 1:**
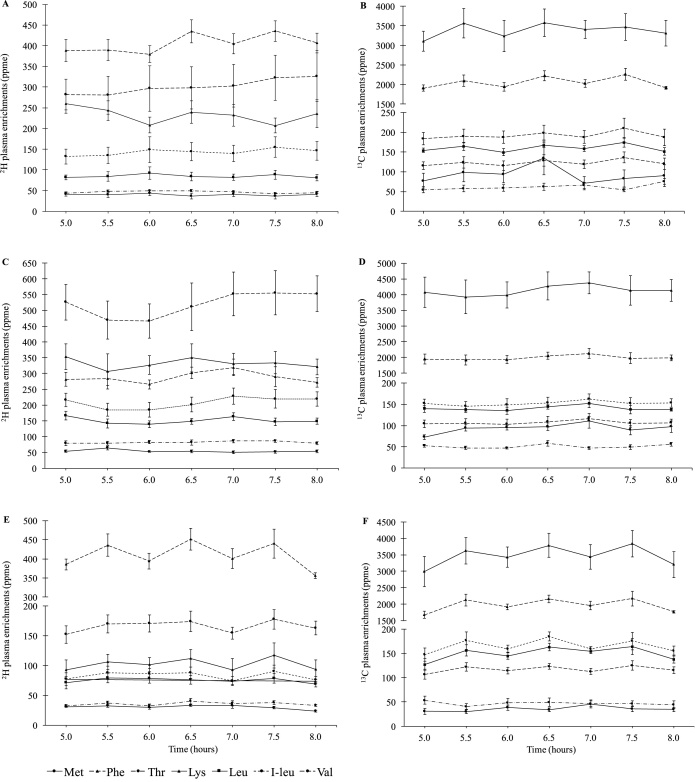
Plasma enrichment of IAA after consumption of different proteins, with different subjects (*n* = 6) in each test protein group. Plasma appearance of (A) ^2^H and (B) ^13^C isotopic enrichments of IAA (ppme) at plateau after consumption of intrinsically labeled egg white protein; plasma appearance of (C) ^2^H and (D) ^13^C isotopic enrichments of IAA (ppme) at plateau after consumption of intrinsically labeled whole boiled egg; and plasma appearance of (E) ^2^H and (F) ^13^C isotopic enrichments of IAA (ppme) at plateau after consumption of intrinsically labeled cooked meat. Plots represent means ± SEs of ^2^H and ^13^C isotopic enrichments appearance in plasma. IAA, indispensable amino acid; ppme, parts per millioin excess.

The plasma ^2^H and ^13^C isotopic enrichments reached a plateau from hour 5 onwards in each experiment and are represented in **Figure 1A–F**. The intensities for methionine and threonine enrichment were low in the plasma for the 3 test proteins. The breath ^13^CO_2_ enrichment is shown in **Supplemental Figure 3** and the meal ^2^H and ^13^C enrichments are provided in **Supplemental Table 1**. The average true ileal IAA digestibility of boiled lyophilized hen's egg white protein, whole boiled egg, and cooked meat is summarized in **Table 4**.

**TABLE 4:** True ileal digestibility values for IAA from boiled hen's egg white, whole egg, and meat protein in healthy adult volunteers^1^

	True ileal digestibility, %		
IAAs	EWP	WBE	CM
Methionine	79.8 ± 4.6	85.9 ± 6.2	92.7 ± 2.2
Phenylalanine	93.0 ± 0.9	95.6 ± 2.1	94.4 ± 1.9
Threonine	88.7 ± 4.6	96.2 ± 2.0	93.7 ± 1.5
Lysine	88.8 ± 3.1	88.9 ± 4.5	95.5 ± 1.2
Leucine	87.9 ± 5.9	87.6 ± 2.4	89.1 ± 4.0
Isoleucine	83.5 ± 6.0	85.4 ± 3.0	88.8 ± 4.1
Valine	82.1 ± 5.6	86.6 ± 5.3	89.6 ± 2.1
Mean IAA	86.3 ± 4.6	89.4 ± 4.5	92.0 ± 2.8

^1^Values are mean percentages ± SDs, *n* = 6/group. Different subjects were used for each experiment. CM, cooked meat; EWP, egg white protein; IAA, indispensible amino acid; WBE, whole boiled egg.

**FIGURE 2:**
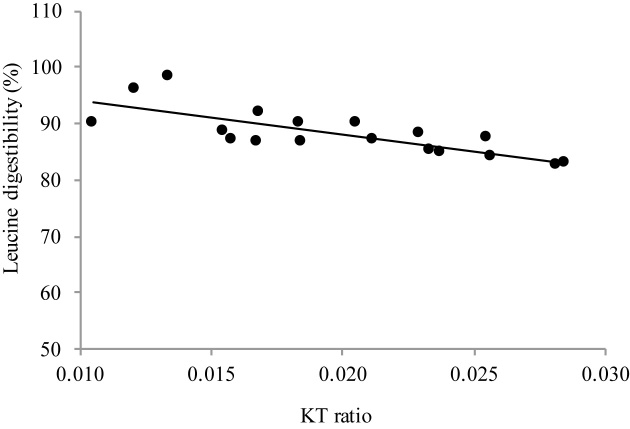
Relation between leucine digestibility and KT ratio of all subjects (*n* = 18). Pearson's *r* = −0.772, *P* = 0.009. KT, kynurenine-to-tryptophan.

The mean ± SD true ileal IAA digestibility was 86.3% ± 4.6% for egg white protein, 89.4% ± 4.5% for whole boiled egg, and 92.0% ± 2.8% for cooked meat. The true ileal IAA digestibility of egg white protein ranged from 79.8% (lowest) for methionine to 93.0% (highest) for phenylalanine. For whole boiled egg and meat, isoleucine had the lowest digestibility, at 85.4% and 88.8%, respectively, and the highest IAA digestibility was found for threonine at 96.2% and lysine at 95.5%. The multivariate ANOVA showed that overall IAA digestibility was significantly different between the 3 test protein groups (Wilks' Lambda test, *P* = 0.031). Specifically, the difference lay between the mean IAA digestibility of egg white protein and meat protein (*P* = 0.01). The digestibilities of egg white protein and whole boiled egg were not significantly different from each other. For individual amino acids, methionine (*P* = 0.001) digestibility was significantly lower for egg white protein than for meat; that of threonine was lower for egg white protein than for both whole egg (*P* = 0.002) and meat (*P* = 0.040). Except for methionine and valine (*r* = 0.86, *P* < 0.001), none of the IAA digestibility values correlated with each other across the different proteins (correlation matrix not presented). The mean and individual IAA digestibilities as measured in comparison to the standard protein were also inversely correlated with the KT ratio and plasma tryptophan and kynurenine values. Among different IAA digestibility values, leucine digestibility was significantly and inversely correlated with the plasma KT ratio (**Figure 2**; *r* = −0.772; *P* = 0.009).

The interindividual variability (CV) also varied within a small range for each IAA within each protein. The interindividual variability for digestibility in egg white protein ranged from 1% for phenylalanine to 7% for isoleucine. For whole boiled egg, the interindividual variability ranged from 2% for threonine to 7% for methionine. For meat, the interindividual variability ranged from 1% for lysine to 5% for isoleucine.

## DISCUSSION

In this study, the mean true ileal IAA digestibility, relative to crystalline IAAs, of protein from boiled lyophilized egg white protein, boiled whole egg, and cooked meat was 86%, 89%, and 92% respectively, when specifically tested within a mixed-meal matrix that also contained cereal, as is the norm for the way these foods are eaten locally. In general, these values were reasonably close to, or slightly lower (within 5–10%) than, reported literature values of protein digestibility measured by ileal or fecal methods, as well as by different meal-processing methods, in humans or animal models (**Table 5**). The reported apparent fecal amino acid and fecal nitrogen digestibility values in the literature ranged from 90% to 97% (15, 22, 23), and were similar to values found in the present study. However, when compared with individual apparent fecal amino acid digestibility values of whole egg in humans (23), leucine, isoleucine, valine, and methionine digestibilities from this study were lower, but those of lysine, phenylalanine and threonine were higher; this could be because different methods were used either for the egg preparation or for the digestibility measurements. The mean ileal IAA digestibility of meat in this study (92%) compared well with measurements in dogs (92%) fed chicken breast meat (24). Similar values (∼90%) were obtained for human true ileal total nitrogen digestibility of beef meat protein (another high-quality protein) under similar cooking conditions (25, 26). Lyophilization of egg white protein has been shown to reduce its digestibility by ∼10% (27); this study found that whole boiled egg protein mean IAA digestibility was slightly, but not significantly, higher than that of boiled lyophilized egg white protein (89.4% compared with 86.3%).

**TABLE 5:** Comparison of digestibility values from adult humans or animal models by use of different methods of preparation and digestibility measurement with values from this study for the 3 test proteins

Test protein	Animal model	Method of preparation	Digestibility method	Mean IAA^1^ digestibility value,^2^ %	Reference
Egg					
Egg white	Human	Boiled for 20 min and lyophilized (hen's egg)	Dual isotope tracer	86.3 ± 4.6	Present study
		Raw lyophilized (quail egg)	In vitro	74.5 ± 0.2	(22)
Whole egg	Human	Boiled for 20 min	Dual isotope tracer	89.4 ± 4.5	Present study
		Microwaved	True ileal digestibility of (leucine)	91	(16)
			Apparent orofecal amino acid digestibility	90.0 ± 2.3	(22)^2^,^3^
			Apparent orofecal amino acid digestibility	92.8 ± 1.7	(23)^2^,^3^
		Spray dried	True orofecal nitrogen digestibility	92–97	(15)
Meat					
Chicken	Human	Pressure cooked	Dual isotope tracer	92.0 ± 2.8	Present study
	Dog	Dried at 71°C and ground	True ileal amino acid digestibility	91.7	(24)
Beef	Human	Fried at 215°C	True ileal nitrogen digestibility	89.6 ± 0.5	(26)
		Steamed at 90°C (30 min)	True ileal nitrogen digestibility	90.1 ± 2.1	(25)
	Pig	Water bath at 95°C and minced	True ileal nitrogen digestibility	95.1 ± 0.7	(28)
		Heated on hot plate, minced and boiled at 80°C	Apparent ileal amino acid digestibility	89.4 ± 0.56	(29)
Pork	Human		Apparent orofecal amino acid digestibility	92.41 ± 1.4	(21)^2^,^3^

^1^IAA, indispensable amino acid.

^2^Method of preparation was not reported.

^3^Values are calculated means of indispensable amino acids as reported in the present study.

The KT ratio reflects either the activity of tryptophan 2,3-dioxygenase in the liver, which is constitutional, or indoleamine 2,3-dioxygenase enzyme, which is induced by interferon-γ in the dendritic cells of the small intestinal epithelium (30) and has been found to be elevated in children with increased gut permeability estimated with the dual-sugar absorption test (31). The kynurenine and KT ratios obtained in the present study fell within the range of normality, hence it is likely that the subjects studied, who were healthy and from a middle socioeconomic stratum, had no significant intestinal dysfunction as estimated by the KT ratio. The apparently normal KT ratios limit the possibility of testing the relation between an impaired gut and digestibility, but since the normality of intestinal function was based on a theoretical cutoff derived from kynurenine and tryptophan concentrations in healthy US subjects (21), a Pearson's correlation was performed to evaluate if, even within this range of normal KT ratios, a relation might exist with animal protein IAA digestibility relative to that of spirulina protein. The digestibility of protein is usually taken to be an intrinsic function of the food protein and the matrix in which it is eaten, but it is possible that host intestinal factors might also be relevant. Support for this approach comes from a study on healthy Indian women, in whom citrulline synthesis rates (possibly reflecting a lower enterocyte mass) were negatively correlated with their intestinal absorptive capacity for sugar, as assessed by urinary mannitol recovery (13). Branched-chain amino acid absorption has also been shown to be lower, by ∼30%, in subjects with tropical sprue, which is similar in intestinal morphological features to EED (32). The specific correlation between postprandial leucine digestibility and the fasting plasma KT ratio might not be directly reflective of digestibility, but instead related to other metabolic effects. For example, leucine has been shown to affect tryptophan metabolism to increase kynurenine either by increasing the activity of tryptophan 2,3-dioxygenase or by inhibiting kynureninase activity, but only at high concentrations (33). To evaluate this better, particularly in a setting where EED might be present, requires further investigation across a range of digestible proteins (not only high-quality proteins) and a wider range of intestinal function. The public health implications of this finding are significant, since undernourished young men and children who may also have a burden of intestinal parasites have been shown to have a higher lysine requirement (34, 35), and a lower splanchnic uptake of leucine (36).

From the different methods proposed by the FAO expert working group, ileal digestibility as determined with the dual stable isotope approach seems to be appropriate and suitable for use across populations, as it meets the challenges associated with measuring true ileal amino acid digestibility in humans, offers measurements of different IAA ileal digestibility, and is minimally invasive in nature (4). The method depends on simultaneous administration of differentially labeled protein sources, one of them acting as a standard, with known digestibility. It is worth emphasizing that in this method, the digestibility of egg and meat protein was determined with reference to a standard protein (spirulina), which in turn was indexed to a crystalline IAA mixture. Since the test protein digestibility is calculated as the postprandial ratio of the appearance, at plateau, of the differentially labeled amino acids in the blood in comparison to their ratio in the test meal, it would minimize the effect of splanchnic handling and post-absorptive metabolism of each amino acid if they were present. This method reports IAA appearance at the site of protein and peptide hydrolysis, that is, the ileum and enterocytes. The same approach has been used in an earlier study as a proof of concept to measure legume protein digestibility (5).

There have been successful efforts at labeling amino acids in hen's egg protein by use of a uniformly ^13^C- and ^15^N-^13^C-labeled amino acid mixture (17, 37, 38) or spirulina (39); however, these studies either did not demonstrate their use in humans or digestibility was not measured. In addition, the dose of the labeled amino acid mixtures used in these earlier studies was higher, ranging from 50 to 400 mg/d. Although the intrinsic labeling of egg protein by use of ^15^N-labeled amino acids has been attempted previously (16, 37), the use of ^15^N for intrinsic labeling was not considered here, as the α-amino group is lost through transamination and deamination, and enters into different nitrogen pools. The hens were dosed orally with the dose mixed in water, which has been shown to achieve better enrichments, probably owing to increased bioavailability when compared with the dose mixed in the feed (17), as well as minimal isotope spillage.

The strength of this study is the use of intrinsically labeled hen egg and meat protein in the dual tracer method to measure true ileal IAA digestibility in humans, along with low interindividual variability. Additionally, measurements of intestinal function were also correlated with digestibility, but there was not a wide range of functional impairment for better evaluation. Limitations of this study include the relatively small number of subjects studied. However, there was minimal variability between subjects. Further, the method needs to be formally validated by statistical comparisons with direct methods (oroileal disappearance) in humans. Nevertheless, the method does have considerable application in human nutrition in different physiological states.

In conclusion, this study demonstrates the feasibility of producing uniformly ^2^H-labeled hen egg and meat protein that can be used to evaluate their true ileal IAA digestibility by the dual isotope tracer approach, in humans. The digestibility values obtained were similar to other published human and animal ileal digestibility studies that used egg and meat protein. The Digestible Indispensable Amino Acid Score for assessing protein quality can be computed from the IAA digestibility values obtained for each test protein from this study.

## Supplementary Material

nqy178_Supplemental_Files

## Notes

This research work was supported by The Wellcome Trust/DBT India Alliance through the Margdarshi fellowship to AVK.

Supplemental Figures 1–3 and Supplemental Table 1 are available from the “Supplementary data” link in the online posting of the article and from the same link in the online table of contents at https://academic.oup.com/ajcn/.

SK and NS contributed equally to this work.

Abbreviations used: EED, environmental enteric dysfunction; IAA, indispensable amino acids; KT, kynurenine-to-tryptophan ratio; LC-MS/MS, liquid chromatography-tandem mass spectrometry; ppme, parts per million excess.
