# Supplementary material for: Ileal digestibility of intrinsically labeled hen's egg and meat protein determined with the dual stable isotope tracer method in Indian adults
Source: Am J Clin Nutr. 2018 Oct 1;108(5):980–7. doi: 10.1093/ajcn/nqy178 (PMC6250983; doi:10.1093/ajcn/nqy178)
Supplement: nqy178_Supplemental_Files [file nqy178_supplemental_files.docx]

Ileal digestibility of intrinsically labeled egg and meat protein measured with the dual stable isotope tracer method in Indian adults

Sindhu Kashyap, Nirupama Shivakumar, Aneesia Varkey, Rajendran Duraisamy, Tinku Thomas , Thomas Preston, Sarita Devi, Anura V Kurpad

Supplemental Table 1. Meal enrichments (ppm excess) of ^2^H and ^13^C in egg white, whole egg and meat protein test meal. ^1^

| Amino Acids | EWP^2^ | | WBE^3^ | | CM^4^ | |
| --- | --- | --- | --- | --- | --- | --- |
|  | ^2^H | ^13^C | ^2^H | ^13^C | ^2^H | ^13^C |
| Methionine | 361.6 ± 144.2 | 639 ± 145.6 | 469.4 ± 113.8 | 821.9 ± 237.1 | 344.3 ± 48.1 | 444.7 ± 74.0 |
| Phenylalanine | 2345.8 ± 232.3 | 11582.9 ± 810.7 | 2070.7 ± 138.4 | 14342.2 ± 1136.8 | 2992.4 ± 503.7 | 14280.7 ± 2559.9 |
| Threonine | 282.9 ± 70.9 | 381.4 ± 40.1 | 806.4 ± 198.0 | 585.0 ± 129.7 | 307.7 ± 28.3 | 462.4 ± 108.3 |
| Lysine | 1884.3 ± 611.5 | 30167.5 ± 5441.6 | 2876.2 ± 288.3 | 41156.2 ± 3232.5 | 736.0 ± 112.5 | 31551.9 ± 6860.8 |
| Leucine | 440.9 ± 65.6 | 865.1 ± 66.2 | 1107.7 ± 114.9 | 1056.1 ± 112.0 | 458.2 ± 44.5 | 967.4 ± 175.7 |
| Iso-leucine | 1004.3 ± 326.1 | 1355.6 ± 224.4 | 2088.9 ± 482.0 | 1553.1 ± 251.7 | 603.8 ± 141.2 | 1290.0 ± 274.3 |
| Valine | 1395.9 ± 461.6 | 546.1 ± 77.0 | 3189.3 ± 837.3 | 634.0 ± 60.4 | 836.6 ± 70.5 | 616.4 ± 81.2 |

^1^Values are mean ± SD, n=6 for each group, subjects were different in each experiment

^2^EWP: Egg white protein

^3^WBE: Whole boiled egg

^4^CM: Cooked meat

Supplemental Figure 1. Subject screening and enrollment flowchart.

Assessed for eligibility (n=22)

Excluded (n=4)

- Not meeting inclusion criteria (n=4)

Participated in tracer experimental protocol in each of the three test protein groups

Enrolled (n=18)

All subjects completed the study and were included for data analysis

Egg white protein

n=6, male and female (1:1)

Whole boiled egg

n=6, male and

female (1:1)

Cooked meat

n=6, male and

female (1:1)

Supplemental Figure 2. ^2^H isotopic enrichments of lyophilized egg white protein for individual indispensable amino acid from the 6 mg ^2^H crystalline amino acid dosing protocol for first 6 days of dosing and showing the increase in enrichment in the first 3 days of 12 mg dosing; mean ± SD.

Supplemental Figure 3. Breath ^13^CO_2_ enrichment averaged across the test protein groups, egg white protein, whole boiled egg and cooked muscle; mean ± SD.
